# Cross-group neutralization of HIV-1 and evidence for conservation of the PG9/PG16 epitopes within divergent groups of HIV-1

**DOI:** 10.1186/1742-4690-9-S2-P53

**Published:** 2012-09-13

**Authors:** M Braibant, E Gong, J Plantier, F Simon, F Barin

**Affiliations:** 1 Charles Nicolle University Hospital, Rouen, France; 2 St Louis Hospital, Paris, France; 3 Universite François Rabelais, and INSERM U966, Tours, France

## Background

HIV-1 has been classified into 4 groups: M, N, O and P. The aim was to revisit the cross-group neutralization using a highly diverse panel of primary isolates (PI) and human monoclonal neutralizing antibodies (mAb).

## Methods

The panel of viruses included 9 HIV-1 group O PIs, 1 recombinant M/O PI, 1 group N PI, 1 group P PI, 2 group M (subtype B) PIs and the HIV-1 group M adapted strain MN. All the viruses were tested for neutralization in TZM-bl cells, using a panel of sera issued from patients infected by HIV-1 group M viruses (n=11), HIV-1 groups O (n=12) and P (n=1). The mAbs were b12, 2G12, 2F5, 4E10, PG9, PG16, VRC01, VRC03 and HJ16.

## Results

The 12 group O sera neutralized from 1 to 6 group O viruses, and 6 of them cross-neutralized one group M PI. Five of the 10 group M sera cross-neutralized from 4 to 9 group O PIs. The group N and P viruses were neutralized by 1-4 of 12 and 4-5 of 11 sera from groups O and M patients, respectively. The human mAbs did not show any cross-group neutralization, except PG9 and PG16. Two group O PIs were neutralized by both PG9 and PG16, and one group O PI was neutralized by PG9 only. The group N PI was highly sensitive to neutralization by PG9 and PG16. The N-linked glycans at positions 156 and 160 and the cationic residues of strand C of the V1/V2 domain that have been identified as part of the PG9 epitope are conserved among the group N.

## Conclusion

The cross-group neutralization of HIV-1 has been demonstrated. The conservation of the PG9 and PG16 epitopes between groups provides an argument for their relevance as components of a potentially efficient HIV vaccine.

